# Psychological and functional outcomes following a randomized controlled trial of surf and hike therapy for U.S. service members

**DOI:** 10.3389/fpsyg.2023.1185774

**Published:** 2023-06-08

**Authors:** Kristen H. Walter, Nicholas P. Otis, Erin L. Miggantz, Travis N. Ray, Lisa H. Glassman, Jessica L. Beltran, Kim T. Kobayashi Elliott, Betty Michalewicz-Kragh

**Affiliations:** ^1^Health and Behavioral Sciences, Naval Health Research Center, San Diego, CA, United States; ^2^Leidos, Inc., San Diego, CA, United States; ^3^Department of Public Health, Naval Medical Center San Diego, San Diego, CA, United States

**Keywords:** major depressive disorder, exercise, nature exposure, nature-based recreation therapy, natural environment, military, outdoor activity

## Abstract

**Introduction:**

Exercise-based interventions have established benefits for the treatment of depression and other psychological outcomes; however, limited data exist evaluating psychological, social, and functional outcomes for exercise outdoors.

**Methods:**

The current study sought to expand knowledge about the breadth of effects following outdoor exercise interventions by using data from a randomized control trial comparing Surf and Hike Therapy among 96 U.S. active duty service members with major depressive disorder (MDD). Assessments examining psychological symptoms and functioning were completed before and after the 6-week programs, and 3 months following program completion. Participants also completed assessments before and after each exercise session. Multilevel modeling was used to determine whether psychological and functional outcomes (anxiety, positive and negative affect, resilience, pain, and physical and social functioning) improved for service members receiving Surf or Hike Therapy, and whether improvements differed by intervention.

**Results:**

Study findings showed improved anxiety (*p* < 0.001), negative affect (*p* < 0.001), psychological resilience (*p* = 0.013), and social functioning (*p* < 0.001) following program participation, with no differences by intervention. Positive affect, pain, and physical functioning did not significantly improve after the program. Within sessions, positive affect (*p* < 0.001) and pain (*p* = 0.036) changed, and to a greater extent for those in the Surf Therapy condition.

**Conclusion:**

Study results suggest that both Surf Therapy and Hike Therapy can improve psychological symptoms and social functioning impairments that commonly co-occur among service members with MDD, but Surf Therapy may provide enhanced immediate effects on positive affect and pain.

**Clinical trial registration:**

ClinicalTrials.gov, NCT03302611.

## 1. Introduction

Many US active duty service members are diagnosed with psychological disorders or experience mental health symptoms, such as posttraumatic stress, alcohol misuse, anxiety, depression, and insomnia (Hourani et al., 2006; Gehrman et al., 2013; Meadows et al., 2021). Physical health problems are also common (Meadows et al., 2021) and may co-occur with psychological symptoms (Boyko et al., 2010; Knapen et al., 2015; Dunbar et al., 2022). These health concerns collectively impact service member functioning, while also increasing rates of absenteeism, health care utilization, and military attrition (Tanielian et al., 2008; Blakeley and Jansen, 2013; Meadows et al., 2021; Dunbar et al., 2022). Finding optimal treatments and adjunctive interventions is imperative to relieve symptoms and improve service member functional outcomes and operational readiness.

Across various populations, established literature and meta-analyses have demonstrated the effects of exercise-based interventions as both primary and adjunctive care for depression outcomes (Craft and Landers, 1998; Robertson et al., 2012; Cooney et al., 2013; Silveira et al., 2013; Rosenbaum et al., 2014; Kvam et al., 2016; Schuch et al., 2016; Stubbs et al., 2016). Although emerging literature supports the use of exercise-based interventions to improve other psychological outcomes [e.g., posttraumatic stress disorder (PTSD) or anxiety], such outcome work is more nascent for outdoor exercise, which is important given that exercise in natural environments may provide enhanced psychological benefit when compared with exercise performed indoors (Thompson Coon et al., 2011). For example, hiking/walking in nature has demonstrated greater improvements in depression (Morita et al., 2007; Li et al., 2008) and related symptoms (Niedermeier et al., 2017; Koselka et al., 2019) compared with walking in an indoor or urban area. Research in various populations has shown that outdoor exercise interventions are associated with improved mental health outcomes, such as reductions in tension, anger, hostility, hopelessness, and suicidal ideation (Morita et al., 2007; Lundberg et al., 2011; Thompson Coon et al., 2011; Sturm et al., 2012; Frumkin et al., 2017). Within military populations, outdoor exercise interventions demonstrated benefits for psychological health, including increased positive affect and decreased negative affect, anxiety, and PTSD symptoms (e.g., Gelkopf et al., 2013; Rogers et al., 2014; Crawford, 2017; Townsend et al., 2018; Greer and Vin-Raviv, 2019; Walter et al., 2019; Bettmann et al., 2021; Littman et al., 2021; Walter et al., 2021). Although research on outdoor exercise interventions for overall psychological health is growing, less is known about the effects of outdoor exercise on physical and social functioning, which are essential for operational readiness and effectiveness.

Given that physical activity is inherent in exercise-based interventions, exploring the effects on physical health and functioning expands knowledge about the extent of the benefits these interventions yield. Among civilian populations, exercise-based interventions were found to improve general physical and global functioning (Mota-Pereira et al., 2011; Gelkopf et al., 2013; Knapen et al., 2015; Poulsen et al., 2018), energy (Morita et al., 2007; Thompson Coon et al., 2011), and functional exercise capacity (Rosenbaum et al., 2014). In addition, research among veterans found reductions in somatic symptoms following outdoor exercise interventions (Vella et al., 2013). However, it is unclear whether outdoor exercise interventions impact physical functioning, which is particularly important among service members because they are required to maintain minimum requirements for physical fitness and often have physical requirements as part of their occupational duties.

The social nature of group-based outdoor exercise has been purported to be an active therapeutic ingredient in these interventions that is supported by qualitative research (Caddick et al., 2015; Marshall et al., 2020). Furthermore, quantitative research studies support enhanced social engagement and support in various populations following outdoor exercise interventions (Lundberg et al., 2011; Mowatt and Bennett, 2011; Thompson Coon et al., 2011; Mason and Holt, 2012; Carless et al., 2013; Gelkopf et al., 2013; Hignett et al., 2018; Britton et al., 2020). In military samples, increased social engagement following exercise-based interventions has been linked to improvements in mood (Carless et al., 2013) and perceived reductions in isolation (Marshall et al., 2020). Social connection may be a key aspect of group-based exercise interventions for military populations. In fact, a scoping review found that the most prominent theme among qualitative studies on exercise-based interventions for veterans was enjoyment of the social aspect of the activity and reconnection with the military community (Shirazipour et al., 2019). The social aspect of group-based outdoor exercise may be particularly beneficial for service members with major depressive disorder (MDD), as loss of interest in activities and socialization is a symptom of the disorder. Furthermore, if service members have duty limitation due to their MDD, they may have fewer opportunities for their usual physical activity and social interaction. Therefore, group-based, outdoor exercise interventions may serve a critical role in improving psychological, physical, and functional outcomes and facilitating recovery among service members with MDD.

Taken together, research strongly supports the use of exercise-based interventions to improve depression and other psychological outcomes across various populations, although there is a gap in comparable literature for outdoor exercise. Recent work adds support for outdoor exercise in improving general psychological and functional outcomes; however, there are several methodological limitations in this literature, including the infrequent use of control groups, absence of multimodal assessment with standardized, validated measures, and scant data evaluating the longer-term effects of interventions. Furthermore, research on exercise and outdoor exercise interventions in military populations focuses primarily on veterans, and fewer data exist examining the effectiveness of these interventions in active duty service members. These methodological concerns make it difficult to isolate the effects of outdoor exercise interventions, determine unique outcomes by intervention type, and identify populations for which these interventions are most effective—all critical elements to inform how these therapies can be used to address psychological and functional concerns.

The current study examined the effects of Surf and Hike Therapies on psychological and functional outcomes among US active duty service members with MDD. Results from the parent study demonstrated that service members who received either Surf or Hike Therapy reported statistically and clinically significant reductions in depression symptom severity and MDD diagnosis (Walter et al., 2023). The current work extends these findings by evaluating whether service members who received either Surf or Hike Therapy also reported improvements in secondary outcomes, including positive and negative affect, pain, anxiety, resilience, and physical and social functioning. Furthermore, this study explores whether Surf and Hike Therapies have differential effects on these outcomes. Our primary hypothesis for the parent study was that Surf Therapy would yield increased benefits compared with Hike Therapy, consistent with the concept of “blue space,” which posits that enhanced effects of exercise interventions occur in or near water (e.g., Britton et al., 2020). However, apart from MDD remission status at 3-month follow-up favoring Surf Therapy, there were no significant differences in primary outcomes between the intervention groups. For this reason, we expected that both interventions would result in significant improvements in psychological symptoms and functioning, and that these benefits would not differ by intervention.

## 2. Methods

### 2.1. Participants

Participants (*N* = 96) were active duty service members who were referred to the Naval Medical Center San Diego (NMCSD) Wounded, Ill, and Injured (WII) Wellness Program between January 2018 and March 2020. Service members who met MDD diagnostic criteria as determined by the Mini International Neuropsychiatric Interview version 7.0 (MINI-7; Sheehan et al., 1998) were eligible for study participation. To control for the dose of intervention, individuals who previously participated in the Surf or Hike Therapy programs were excluded from study participation; this was the only exclusion criterion. To balance groups during recruitment, a 10-block randomization scheme was used to randomly assign eligible participants to either the Surf or Hike Therapy. Although concurrent engagement in psychotherapy or use of prescribed psychotropic medications were not exclusion criteria for this study, data on these variables were collected and analyzed. Considering the military’s transitory nature, participants who wished to take part in the nonrandomized study intervention were able to do so during their follow-up period, and data were also collected on this participation, if applicable.

**Figure 1:**
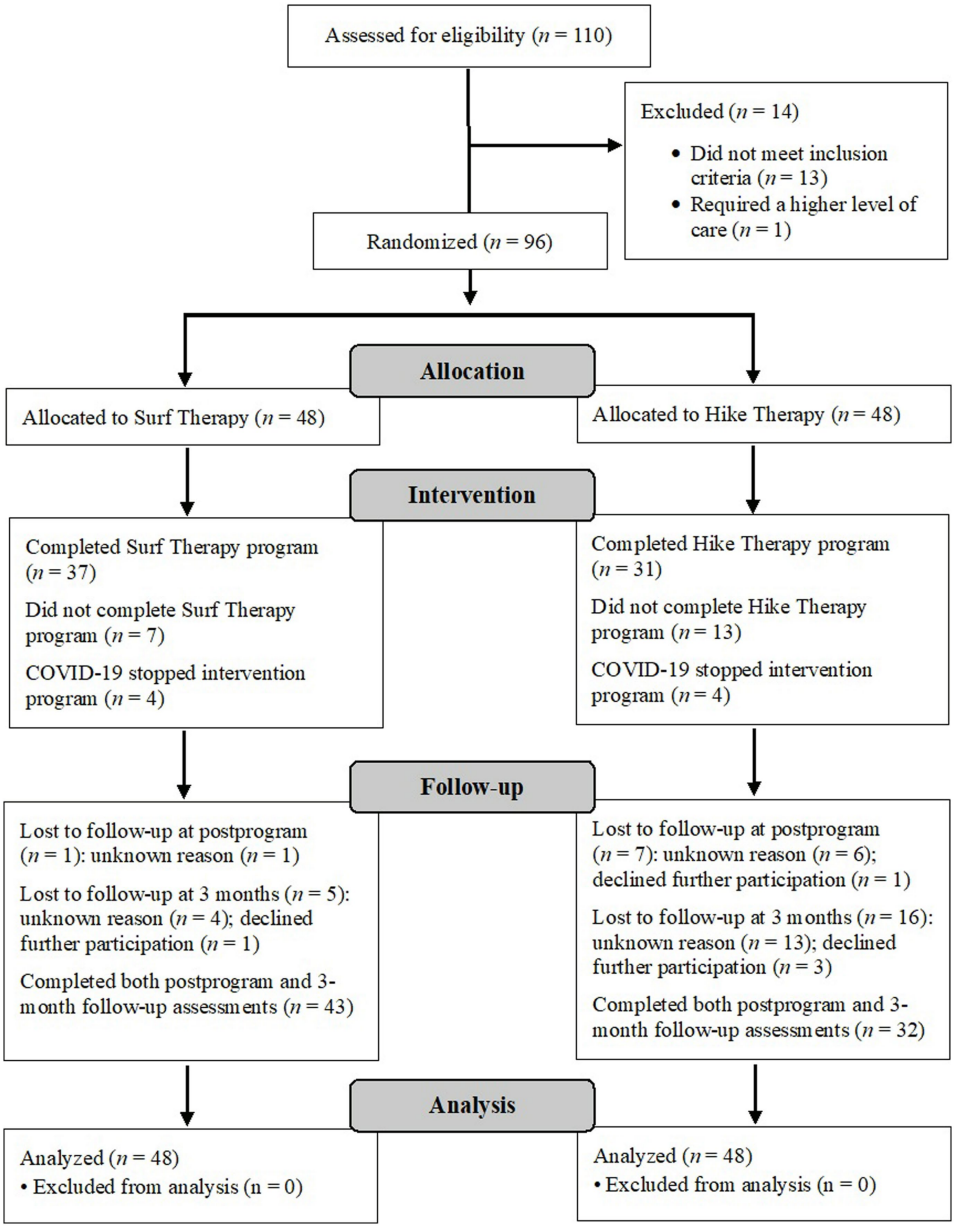
CONSORT flowchart of participants. Intervention “completion,” as determined by the NMCSD programs, was defined as completing all but two of the available sessions (up to six sessions) for each modality. The programs were halted due to the COVID-19 pandemic; participants in this cohort (*n* = 8) were counted neither as completers nor non-completers. Their data were analyzed as intent-to-treat. Total lost to follow-up is greater than the total number allocated because many participants completed either the postprogram assessment or the follow-up assessment and are thus counted twice. RCT, randomized controlled trial; COVID-19, coronavirus disease 2019.

Study participants were primarily white (42%) with a mean age of 28 years. Most participants received concurrent pharmacotherapy for a mental health concern (71%) and were moderately or highly physically active according to the International Physical Activity Questionnaire-Short Form (39 and 33%, respectively; IPAQ-SF; Craig et al., 2003; IPAQ Research Committee, 2005). Most participants completed their randomized intervention (77%), and 99% of available session assessments were completed. Trauma exposure was common in this sample; 77% of participants reported exposure to a PTSD Criterion A traumatic event. The most frequently reported traumatic events included sexual assault (47%), combat (11%), and assault with a weapon [8%; based on the extended Life Events Checklist (LEC); Weathers et al., 2013a]. In addition to a diagnosis of MDD, 60% of the sample met diagnostic criteria for PTSD (*n* = 58; Surf = 24, Hike = 34). For further demographic information, see Table 1, and for a study CONSORT diagram, see Figure 1.

**Table 1:** Preprogram sample characteristics.

Characteristic	Total sample (*N* = 96)	Surf (*n* = 48)	Hike (*n* = 48)
	*n* (%)	*n* (%)	*n* (%)
Race/ethnicity^a^			
Asian or Asian-American & Native American or Alaska Native	4 (4.2)	–	–
Black or African American	15 (15.6)	–	–
Hispanic, Latino, or Spanish origin	18 (18.8)	–	–
Multiracial	19 (19.8)	–	–
White	40 (41.7)		
Gender identity			
Men	46 (47.9)	26 (54.2)	20 (41.7)
Women	50 (52.1)	22 (45.8)	28 (58.3)
Rank^a^			
E1–E4	34 (35.4)	–	–
E5–E9	57 (59.4)	–	–
Officer	5 (5.2)	–	–
Concurrent mental health treatment	89 (92.7)	44 (91.7)	45 (93.8)
Pharmacotherapy	68 (70.8)	34 (70.8)	34 (70.8)
Psychotherapy	89 (92.7)	44 (91.7)	45 (93.8)
Activity level^b^			
Low/inactive	10 (10.4)	8 (16.7)	2 (4.2)
Moderately active	37 (38.5)	18 (37.5)	19 (39.6)
Highly active	32 (33.3)	13 (27.1)	19 (39.6)
Completion of assigned program^c^^,^^d^	68 (77.3)	37 (84.1)	31 (70.5)
	*M* (*SD*)	*M* (*SD*)	*M* (*SD*)
Age, years	28.1 (5.6)	29.3 (6.2)*	27.0 (4.8)*
Education, years	13.2 (1.7)	13.3 (1.7)	13.2 (1.7)
Sessions attended^d^^,^^e^	3.9 (1.6)	3.9 (1.3)	4.0 (1.9)
Yoga sessions attended	0.7 (1.2)	1.3 (1.4)	–
Preprogram measures			
GAD-7	13.8 (5.1)	12.8 (4.8)	14.8 (5.2)
PAS	20.6 (7.0)	22.4 (7.7)**	18.6 (5.8)**
NAS	23.9 (8.0)	21.5 (6.9)**	26.4 (8.4)**
RSES-4	9.6 (3.5)	10.2 (3.4)	9.1 (3.5)
NPRS	3.1 (2.5)	2.9 (2.3)	3.4 (2.6)
SF-36–PF	51.4 (8.1)	51.7 (8.1)	51.1 (8.1)
SF-36–SF	31.6 (8.8)	32.3 (8.1)	31.0 (9.5)
PCL-5^f^	50.3 (13.3)	46.5 (12.7)	52.9 (13.3)

E, enlisted rank; GAD-7, 7-item generalized anxiety disorder scale; NAS, negative affect schedule; NPRS, numerical pain rating scale; PAS, positive affect schedule; PCL-5, PTSD checklist for DSM-5; RSES-4, 4-item response to stressful events scale; SF-36–PF, 36-item short form health survey, version 2: physical functioning subscale; SF-36–SF, 36-item short form health survey, version 2: social functioning subscale. Totals may not sum to sample numbers or percentages due to missing data. Asterisks indicate significant difference between activity conditions.

a All attempts were made to report race/ethnicity and rank data properly, but due to low cell counts, variables were combined to protect participant identity, and they are not stratified by condition.

b Physical activity level data were calculated (see Naugle et al., 2012) from the self-report version of the International Physical Activity Questionnaire-Short Form.

c Program completion was defined by the Naval Medical Center San Diego as missing no more than two sessions of the assigned modality.

d Because programs were halted due to the sudden onset of COVID-19, participants (*n* = 8) in the affected cohort were not counted in completion or attendance statistics.

e Included are only sessions in which the assigned modality was conducted. Occasionally, due to adverse weather, sessions consisted of alternative activities (e.g., visit to the National Surf Museum, hiking strength and conditioning class).

f PCL-5 scores range from 0 to 80 and are only reported for those with a diagnosis of PTSD (based on the MINI-7).

^*^p < 0.05; ^**^p < 0.01.

### 2.2. Program

The NMCSD WII program was intended for service members with psychological and/or physical injuries. The program offered Surf and Hike Therapies as options for standard care. Both programs offered sessions that lasted 3–4 h and were conducted once per week for 6 consecutive weeks. Cohorts of about 20 service members were accommodated per cycle. Surf Therapy took place at a public beach in San Diego and Hike Therapy occurred at various locations across San Diego County. The NMCSD program offered an optional yoga practice for participants prior to the start of each Surf Therapy session (data were collected and analyzed). Further information on the Surf Therapy and Hike Therapy programs has been published elsewhere (see Walter et al., 2019, 2023). In early 2020, the NMCSD WII Wellness Program was forced to cancel all in-person group activities due to the COVID-19 pandemic. As a result, participants from the cohort that began in March 2020 were unable to complete the program.

### 2.3. Procedure

Participants were assessed at preprogram, postprogram, and 3-month follow-up, as well as at each session. The preprogram assessment consisted of self-report and clinician-administered measures that evaluated MDD and related symptoms. Assessors determined MDD diagnosis using the MINI-7, a reliable and valid diagnostic assessment across many different psychological conditions (e.g., Sheehan et al., 1997). The assessor was blinded to participants’ intervention condition. Participants began their assigned intervention program within 2 weeks of completing their preprogram assessment. Throughout the 6-week Surf and Hike Therapy programs, participants completed self-report assessments immediately before and after each session. Participants were asked to complete a postprogram assessment within 2 weeks of their last session, as well as a final follow-up assessment conducted 3 months later. The Institutional Review Board at NMCSD approved all study procedures.

### 2.4. Measures

#### 2.4.1. Anxiety

Anxiety symptom severity over the last 2 weeks was assessed using the Generalized Anxiety Disorders 7-item measure (GAD-7; Spitzer et al., 2006). The seven symptom items are rated on a scale from 0 to 3 and summed for a total score, with higher total scores indicating greater severity of anxiety. The internal consistency of the GAD-7 was *α* = 0.86–0.92. The GAD-7 was completed preprogram, postprogram, and at 3-month follow-up.

#### 2.4.2. Affect

Positive and negative affect were measured with the Positive and Negative Affect Schedule (PANAS; Watson et al., 1988). The PANAS consists of 20 items: 10 positive (PAS) and 10 negative (NAS) emotions that are assessed at the current moment. The items were rated from 0 to 4 and summed to create positive and negative affect subscales. Higher scores indicate higher levels of positive and negative affect, respectively. Within this sample, Cronbach alphas ranged from *α* = 0.84–0.94 for the PAS and *α* = 0.84–0.92 for the NAS. The PANAS was completed preprogram, postprogram, and at 3-month follow-up. Only the PAS was completed before and after each session (α = 0.91–0.98) to reduce participant burden and assess positive outcomes to complement the symptom measures.

#### 2.4.3. Resilience

Psychological resilience was measured with the Response to Stressful Events Scale (RSES-4; De La Rosa et al., 2016). This four-item instrument is scored from 0 (*not at all like me*) to 4 (*exactly like me*) and higher sum scores indicate greater resilience in response to stressful events. In this study, internal consistency was *α* = 0.81–0.88. The RSES-4 was completed preprogram, postprogram, and at 3-month follow-up.

#### 2.4.4. Pain

Participants’ current level of pain was measured using the single-item Numerical Pain Rating Scale (NPRS; McCaffery and Beebe, 1989). Participants were asked to rate their current pain level on a scale ranging from 0 (*no pain*) to 10 (*worst imaginable pain*). The NPRS was completed at preprogram, postprogram, 3-month follow-up, and before and after each session.

#### 2.4.5. Physical and social functioning

The Short Form Health Survey-36 Item, Version 2 (SF-36v2; Ware, 2000) was used to measure physical and social functioning. Norm-based scores for an adult U.S. depression sample are produced using the SF-36 software (QualityMetric, Johnston, RI, United States). The SF-36v2 was completed preprogram, postprogram, and at 3-month follow-up.

#### 2.4.6. Physical activity

At preprogram, the frequency and intensity of physical activity occurring over the past 7 days were assessed with the seven-item International Physical Activity Questionnaire-Short Form (IPAQ-SF; Craig et al., 2003). IPAQ-SF scores were calculated in metabolic equivalent minutes (MET mins), which are divided into three categories aligning with the guidelines of the World Health Organization: low (<600 MET mins/week), medium (600–2,999 MET mins/week), and high (≥3,000 MET mins/week).

#### 2.4.7. Posttraumatic stress disorder

Posttraumatic stress disorder diagnosis and severity was assessed at preprogram, postprogram, and 3-month follow-up using the MINI-7 (Sheehan et al., 1998). Self-reported PTSD symptoms were assessed using the PTSD Checklist for DSM-5 (PCL-5; Weathers et al., 2013b) with the extended LEC (Weathers et al., 2013a). In this study, PTSD-related outcomes are reported descriptively.

#### 2.4.8. Data analysis

Data were checked for plausibility and analyzed as intent-to-treat. Descriptive statistics were computed to establish sample and preprogram characteristics. Independent samples *t*-tests and Chi-square tests of association were then used to identify differences in sample characteristics by intervention condition (i.e., Surf vs. Hike Therapy). To examine the change in symptoms, and physical and social functioning over time, separate multilevel models (MLMs) were run for the GAD-7, PAS, NAS, RSES-4, and NPRS. All analyses were conducted in SPSS Version 25 (IBM, Armonk, NY, United States).

Both longitudinal (preprogram, postprogram, and 3-month follow-up) and within-session (presession, postsession) analyses were conducted with MLMs using a step-up model building process. Logical covariance matrices were compared and selected based on model fit according to Akaike information criterion with respect to the number of parameters specified. Additionally, previous work in the parent study (Walter et al., 2023) informed the exclusion of certain predictor variables from final models. All final MLMs used restricted maximum likelihood to account for missing data.

For longitudinal analyses, time was a repeated effect of subject with an unstructured covariance matrix. Piecewise analysis was used with longitudinal MLMs to best account for differing independent variables in the intervention and follow-up periods (e.g., treatment use during each time frame). Fixed effects were specified as follows: time (0 = Postprogram, 1 = Preprogram in pre- to postmodels; 0 = 3-month follow-up, 1 = Postprogram in follow-up models), intervention condition (0 = Hike Therapy, 1 = Surf Therapy), concurrent pharmacotherapy for any mental health concern (0 = Yes, 1 = No), and number of exercise therapy sessions attended (continuous total). Each fixed effect was also used in an interaction term with time. The following variables were dropped before final models due to lack of significance here and in the parent study (Walter et al., 2023): concurrent psychotherapy for any mental health concern, physical activity level, and number of yoga sessions attended (continuous total).

For within-session analyses, intercept, time (pre- to postsession), week of session, and a crossed effect of Time × Week of Session were set as random slopes by subject with a first-order autoregressive covariance matrix. Time × Week of Session was set as a repeated effect of subject and used a compound symmetry covariance matrix. Fixed effects included time (0 = Postsession, 1 = Presession), intervention condition (0 = Surf Therapy, 1 = Hike Therapy), concurrent pharmacotherapy for any mental health concern (0 = Yes, 1 = No), and week of exercise session (continuous week number). All fixed effects were also used in individual interactions with time. Concurrent psychotherapy for any mental health concern and physical activity level were dropped before final models due to lack of significance.

## 3. Results

A total of 96 service members diagnosed with MDD were enrolled in the study; half were randomized to Surf Therapy (*n* = 48) and half to Hike Therapy (*n* = 48). Significant preprogram differences emerged in positive and negative affect between Surf Therapy and Hike Therapy participants. Specifically, those randomized to Surf Therapy had greater PAS and lower NAS scores. As for other demographic measures, the Surf and Hike Therapy groups did not significantly differ. In addition to a MDD diagnosis, participants reported moderate levels of anxiety, as indicated by an average preprogram GAD-7 score of nearly 14 points (Walter et al., 2023). For further detail on descriptive statistics, see Table 1.

### 3.1. Psychological outcomes

#### 3.1.1. Anxiety

Across participants, GAD-7 scores changed significantly from pre- to postprogram (*MD* = −3.07, *p* < 0.001). Specifically, self-reported anxiety scores decreased from 13.30 to 10.23 points. There were no significant differences between Surf and Hike Therapy on change in anxiety severity from pre- to postprogram (*p* = 0.387). As evidenced by a significant Time × Pharmacotherapy interaction, participants who reported pharmacotherapy treatment for any mental health concern reduced their scores 2.55 points more compared with those who did not (*p* = 0.029). Change in anxiety scores over time were also influenced by the number of sessions attended; a significant Time × Session interaction showed that for each additional session attended, GAD-7 scores decreased by 0.60 points (*p* = 0.043). GAD-7 scores did not significantly change from postprogram to 3-month follow-up (*p* = 0.615). For a complete list of longitudinal program results, see Table 2.

**Table 2:** Mean differences from estimated marginal means of final multilevel models examining psychological outcomes over time.

	Pre- to postprogram				Postprogram to 3-month follow-up	
Variable	*MD*	95% CI	*p*	*MD*	95% CI	*p*
	GAD-7					
Time	−3.07	[−4.21, −1.93]	**<0.001**	−0.68	[−3.42, 2.05]	0.615
Intervention condition	−2.41	[−4.44, −0.38]	**0.020**	−1.65	[−5.33, 2.03]	0.369
Time × Intervention condition	−0.89	[−2.91, 1.14]	0.387	−0.67	[−4.61, 3.27]	0.732
Pharmacotherapy	1.22	[−1.02, 3.47]	0.282	6.29	[1.93, 10.64]	**0.006**
Time × Pharmacotherapy	−2.55	[−4.83, −0.27]	**0.029**	−1.11	[−6.32, 4.10]	0.669
Sessions attended	0.08	[−0.47, 0.63]	0.776	0.03	[−1.08, 1.14]	0.956
Time × Sessions attended	−0.60	[−1.19, −0.02]	**0.043**	−0.31	[−1.33, 0.71]	0.542
	PAS					
Time	2.26	[−0.03, 4.56]	0.053	−0.95	[−5.46, 3.56]	0.671
Intervention condition	3.77	[0.94, 6.61]	**0.010**	5.18	[−1.59, 11.96]	0.129
Time × Intervention condition	−0.12	[−4.21, 3.98]	0.955	4.63	[−2.05, 11.30]	0.167
Pharmacotherapy	−0.10	[−3.25, 3.04]	0.948	−2.22	[−10.19, 5.76]	0.577
Time × Pharmacotherapy	3.13	[−1.46, 7.73]	0.178	3.26	[−5.17, 11.70]	0.436
Sessions attended	0.33	[−0.44, 1.10]	0.393	1.41	[−0.39, 3.21]	0.122
Time × Sessions attended	1.01	[−0.16, 2.18]	0.089	0.66	[−1.05, 2.36]	0.438
	NAS					
Time	−3.66	[−5.42, −1.91]	**<0.001**	−2.07	[−5.39, 1.25]	0.213
Intervention condition	−4.41	[−7.48, −1.33]	**0.005**	−1.62	[−6.90, 3.67]	0.538
Time × Intervention condition	0.94	[−2.17, 4.06]	0.548	0.67	[−4.42, 5.76]	0.791
Pharmacotherapy	1.90	[−1.50, 5.31]	0.270	4.75	[−1.41, 10.91]	0.127
Time × Pharmacotherapy	−2.34	[−5.85, 1.16]	0.187	−1.78	[−7.99, 4.43]	0.565
Sessions attended	0.05	[−0.81, −0.91]	0.909	0.98	[−0.76, 2.73]	0.258
Time × Sessions attended	−1.19	[−2.08, −0.30]	**0.010**	−0.81	[−2.13, 0.51]	0.221
	RSES-4					
Time	0.98	[0.21, 1.76]	**0.013**	0.39	[−1.03, 1.82]	0.581
Intervention condition	0.59	[−0.71, 1.89]	0.370	−0.02	[−2.30, 2.27]	0.989
Time × Intervention condition	−1.05	[−2.43, 0.34]	0.136	1.47	[−0.71, 3.64]	0.179
Pharmacotherapy	0.50	[−0.94, 1.94]	0.494	1.07	[−1.60, 3.73]	0.423
Time × Pharmacotherapy	0.98	[−0.56, 2.52]	0.209	0.20	[−2.47, 2.86]	0.883
Sessions attended	0.05	[−0.34, 0.45]	0.795	0.06	[−0.67, 0.80]	0.862
Time × Sessions attended	0.09	[−0.31, 0.48]	0.655	0.12	[−0.44, 0.68]	0.669

MD, mean difference; GAD-7, generalized anxiety disorder scale; NAS, negative affect schedule; PAS, positive affect schedule; RSES-4, 4-item response to stressful events scale. Significant values are bolded. For Intervention Condition, Surf Therapy is the comparator group; for Pharmacotherapy, those who reported psychiatric pharmacotherapy use were the comparator group. Pharmacotherapy use is relative to each time point and was used as a predictor; for pre- to postprogram, preprogram usage was utilized; for postprogram to follow-up, postprogram usage was utilized. Similarly, the sessions attended variable represents those from pre- to postprogram and postprogram to follow-up, respectively, if applicable.

#### 3.1.2. Positive affect

Overall, PAS scores did not significantly improve from pre- to postprogram (*MD* = 2.26, *p* = 0.053) or from postprogram to 3-month follow-up (*p* = 0.671; see Table 2). However, on average, PAS scores significantly increased within each session, as evidenced by a significant main effect of Time from pre- to postsession (*MD* = 8.38, *p* < 0.001). Specifically, participants reported average scores of 24.29 and 32.67 at the start and end of sessions, respectively. This improvement over time was influenced by program type; a significant Time × Intervention Condition interaction showed that participants in Surf Therapy improved 2.80 points more over a session compared with those in Hike Therapy (*p* = 0.012). For a complete list of session results, see Table 3.

**Table 3:** Mean differences from estimated marginal means of final multilevel models examining positive affect and pain within sessions.

Variable	MD	95% CI	*p*
	PAS		
Time (pre- to post-session)	8.38	[7.10, 9.65]	**<0.001**
Intervention condition	5.77	[2.75, 8.78]	**<0.001**
Time × Intervention condition	2.79	[0.65, 4.94]	**0.011**
Pharmacotherapy	−1.51	[−4.86, 1.84]	0.373
Time × Pharmacotherapy	1.10	[−1.26, 3.46]	0.359
Week of session	−0.26	[−0.82, 0.31]	0.370
Time × Week of session	0.42	[−0.27, 1.11]	0.232
	NPRS		
Time (pre- to post-session)	0.23	[0.02, 0.45]	**0.036**
Intervention condition	0.05	[−0.89, 1.00]	0.913
Time × Intervention condition	−0.71	[−1.09, −0.34]	**<0.001**
Pharmacotherapy	−0.90	[−1.95, 0.15]	0.092
Time × Pharmacotherapy	−0.10	[−0.52, 0.31]	0.622
Week of session	−0.03	[−0.14, 0.07]	0.535
Time × Week of session	0.10	[−0.03, 0.22]	0.124

MD, mean difference; NPRS, numerical pain rating scale; PAS, positive affect schedule. Significant values are bolded. For intervention condition, Surf Therapy is the comparator group; for Pharmacotherapy, those who reported psychiatric pharmacotherapy use were the comparator group.

In prior work (Walter et al., 2023), the Surf and Hike Therapy groups significantly differed on self-reported depression severity at preprogram; here, the same groups significantly differed on preprogram positive and negative affect, which are constructs related to depression. For this analysis, to ensure that the observed positive affect differences between Surf and Hike Therapy over the course of a session were not merely a product of these depression differences between groups, we examined Preprogram Depression Severity × Time interactions *post-hoc*. In models without Intervention Condition, both clinician-rated (Montgomery-Åsberg Depression Rating Scale; Montgomery and Åsberg, 1979, *p* = 0.331) and self-reported (Nine-item Patient Health Questionnaire; Kroenke et al., 2001, *p* = 0.250) depression severity were nonsignificant in their respective Symptom Severity × Time interactions, indicating that PAS change over time was likely not a product of preprogram depression severity, but instead, related to intervention condition.

Because some Surf Therapy participants chose to attended yoga prior to their session (representing 14% of the available sessions across both intervention conditions), a subanalysis was also run to examine the impact of yoga session attendance on within-session positive affect scores. A Time × Yoga Attendance interaction was nonsignificant (*p* = 0.934), indicating that the amount of change in positive affect following a session was not related to yoga attendance.

#### 3.1.3. Negative affect

Significant reductions in reported NAS scores were noted among participants from pre- to postprogram, as evidenced by a significant main effect of Time (*MD* = −3.66, *p* < 0.001). On average, participants’ scores decreased from 23.26 to 19.60 points. There was no significant difference between the intervention conditions on change in negative affect from pre- to postprogram (*p* = 0.562). NAS results were influenced by the total number of sessions attended; with each additional session attended, NAS scores decreased by an average of −1.19 points from pre- to postprogram (*p* = 0.010). There was no significant change in NAS scores from postprogram to 3-month follow-up (*p* = 0.213).

#### 3.1.4. Resilience

Resilience scores significantly changed from pre- to postprogram (*p* = 0.013), but not during the follow-up period (*p* = 0.581). The estimate was modest (*MD* = 0.98), such that participants improved from 9.64 to 10.62 points, on average, from pre to postprogram. No predictor variables were significant, including the Time × Intervention Condition interaction (*p* = 0.136; see Table 2).

### 3.2. Functional outcomes

#### 3.2.1. Pain

Pain did not significantly change from pre- to postprogram (*p* = 0.776) or postprogram to 3-month follow-up (*p* = 0.942; see Table 4). Pain increased over the course of a session, as indicated by a main effect of Time (*p* = 0.036), although the estimate was small (*MD* = 0.23). Additionally, a significant Time × Intervention Condition interaction showed that those in Surf Therapy changed by an average of 0.71 points relative to those in Hike Therapy (*p* < 0.001), such that pain scores non-significantly decreased −0.12 points during Surf Therapy (*p* = 0.393) but significantly increased 0.59 points during Hike Therapy sessions (*p* < 0.001). For a complete list of within-session fixed effects, see Table 3.

**Table 4:** Mean differences from estimated marginal means of final multilevel models examining physical and social outcomes over time.

	Pre- to postprogram			Postprogram to 3-month follow-up		
Variable	MD	95% CI	*p*	MD	95% CI	*p*
	NPRS					
Time	0.06	[−0.37, 0.50]	0.776	0.53	[−0.52, 1.58]	0.313
Intervention condition	−0.14	[−1.10, 0.81]	0.769	−0.56	[−2.26, 1.14]	0.508
Time × Intervention condition	0.36	[−0.42, 1.15]	0.357	−0.99	[−2.59, 0.61]	0.216
Pharmacotherapy	−0.87	[−1.93, 0.19]	0.106	−0.46	[−2.44, 1.53]	0.643
Time × Pharmacotherapy	−0.79	[−1.65, 0.08]	0.075	−0.13	[−2.07, 1.80]	0.890
Sessions attended	0.02	[−0.27, 0.30]	0.909	−0.08	[−0.57, 0.41]	0.738
Time × Sessions attended	−0.17	[−0.40, 0.05]	0.127	−0.06	[−0.46, 0.34]	0.763
	SF-36–PF					
Time	−1.55	[−3.12, 0.03]	0.055	−1.07	[−3.81, 1.66]	0.429
Intervention condition	0.91	[−2.07, 3.90]	0.545	1.44	[−3.65, 6.53]	0.568
Time × Intervention condition	0.83	[−2.01, 3.67]	0.562	1.01	[−3.01, 5.04]	0.611
Pharmacotherapy	4.95	[1.65, 8.25]	**0.004**	3.27	[−2.68, 9.21]	0.272
Time × Pharmacotherapy	2.87	[−0.28, 6.03]	0.074	4.22	[−0.89, 9.33]	0.102
Sessions attended	0.39	[−0.51, 1.30]	0.390	0.52	[−0.80, 1.85]	0.429
Time × Sessions attended	0.17	[−0.64, 0.98]	0.680	0.15	[−0.88, 1.18]	0.770
	SF-36–SF					
Time	5.86	[3.51, 8.21]	**<0.001**	−0.05	[−6.24, 6.14]	0.986
Intervention condition	2.78	[−0.92, 6.48]	0.139	4.73	[−2.53, 11.99]	0.194
Time × Intervention condition	2.69	[−1.52, 6.90]	0.208	3.52	[−5.87, 12.91]	0.451
Pharmacotherapy	2.07	[−2.03, 6.17]	0.319	−2.51	[−11.11, 6.08]	0.557
Time × Pharmacotherapy	2.90	[−1.80, 7.60]	0.223	4.68	[−6.91, 16.27]	0.417
Sessions attended	−0.31	[−1.32, 0.69]	0.539	1.22	[−1.09, 3.52]	0.291
Time × Sessions attended	1.98	[0.77, 3.18]	**0.002**	−0.69	[−3.12, 1.74]	0.567

MD, mean difference; NPRS, numerical pain rating scale; SF-36–PF, 36-item short form survey, version 2: physical functioning subscale; SF-36–SF, 36-item short form survey, version 2: social functioning subscale. Significant values are bolded. For intervention condition, Surf Therapy is the comparator group; for pharmacotherapy, those who reported psychiatric pharmacotherapy use were the comparator group. Pharmacotherapy use is relative to each time point and used as a predictor; for pre- to postprogram, preprogram usage was utilized; for postprogram to follow-up, postprogram usage was utilized. Similarly, the sessions attended variable represents those from pre- to postprogram and postprogram to follow-up, respectively, if applicable.

Like PAS, because a small portion of Surf Therapy participants attended yoga before their session, we conducted a subanalysis to examine whether yoga impacted change in NPRS scores over time. A Time × Yoga Attendance interaction was significant (*p* = 0.007, *MD* = −0.74). Specifically, those who attended yoga did not significantly change their pain scores (*MD* = −0.07, *p* = 0.835), but those who did not attend yoga reported an increase in pain of 0.67 points (*p* = 0.011) on an 11-point scale.

#### 3.2.2. Physical functioning

There were no significant effects of time on SF-36 Physical Functioning (PF) scores from pre- to postprogram (*p* = 0.055). There was also no significant difference in change in physical functioning between the intervention conditions from pre- to postprogram (*p* = 0.548). Furthermore, physical functioning did not significantly change from postprogram to 3-month follow-up (*p* = 0.429).

#### 3.2.3. Social functioning

Survey-36 Social Functioning (SF) scores changed significantly from pre- to postprogram (*MD* = 5.86, *p* < 0.001) but not during the follow-up period (*p* = 0.986). The number of sessions attended was significantly related to the change from pre- to postprogram (*p* = 0.002). For each session attended, the SF T-score increased by an average of 1.98 points. The Time × Intervention Condition was not significant from pre- to postprogram (*p* = 0.208), indicating that social functioning improvements did not differ between intervention conditions.

## 4. Discussion

Surf and Hike Therapies integrate several social, psychological, physiological, and environmental elements that may contribute to the alleviation of psychological symptoms, including MDD and PTSD (Thompson Coon et al., 2011; Barton et al., 2012; Rogers et al., 2014; Caddick et al., 2015; Crawford, 2017; Walter et al., 2019, 2023; Otis et al., 2020). However, limited research has examined other outcomes of these interventions, especially within military populations. The current study examined the effects of Surf and Hike Therapies on psychological and functional outcomes in a sample of active duty service members with MDD. Results indicated that both interventions were associated with improved general anxiety symptoms, negative affect, psychological resilience, and social functioning from pre- to postprogram, and these improvements were maintained at the 3-month follow-up assessment. Positive affect, pain, and physical functioning did not statistically improve during this time frame. However, there were within-session changes in positive affect and pain, with service members randomized to Surf Therapy reporting significantly greater benefits on these constructs relative to those randomized to Hike Therapy. These results suggest that both Surf Therapy and Hike Therapy can provide benefits beyond the reduction of depression symptoms in service members with MDD, and that Surf Therapy may provide enhanced *immediate* effects on positive affect and pain.

Results were consistent with prior research reporting postprogram improvements in psychological and functional outcomes for outdoor exercise and recreation programs (Lundberg et al., 2011; Duvall and Kaplan, 2014; Rogers et al., 2014; Bennett et al., 2017; Crawford, 2017; Townsend et al., 2018; Walter et al., 2019, 2021, 2023). Though, in the current study, improvements in negative affect and social functioning were contingent on the number of sessions attended, with a greater number of sessions yielding stronger effects. Reductions in general anxiety symptoms also were reliant on concurrent enrollment in pharmacotherapy, consistent with findings for self-reported depression symptoms in the parent study (Walter et al., 2023). More specifically, participants who reported pharmacotherapy use for any mental health concern reported greater reductions in anxiety symptoms. Collectively, these results suggest that there may be “dose effects” of Surf and Hike Therapies, wherein engagement in the activities on repeated occasions is necessary to produce their intended effects. Results also provide evidence for the notion that Surf and Hike Therapies are suitable adjunctive treatments to conventional treatments (i.e., psychotherapy, pharmacotherapy). More research is needed, however, to determine whether exercise-based interventions can function as stand-alone treatments.

Of the outcomes explored in this study, only physical functioning did not significantly change over the course of the interventions. Several factors may have contributed to the lack of significant findings for physical functioning. Approximately 72% of study participants were at least moderately active at the preprogram assessment. Participants were also seeking an outdoor exercise intervention as part of their standard medical care. Additionally, all participants were active duty service members who must meet minimum physical activity requirements to be considered fit for duty, so it is possible that there was a ceiling effect for this sample that made it difficult to significantly increase physical functioning. Although positive affect changed during a session, it did not over the course of the programs. Previous work by Walter et al. (2019, 2021) showed that positive affect significantly improved before and after outdoor activity programs; however, diverging findings may be due to methodological differences. For example, in the prior studies, the postprogram assessment data were collected on the same day as the final session, which may have conflated program effects with that day’s session effects. In the current study, postprogram assessments were completed within 2 weeks of program end, thereby eliminating this potential confound. Similarly, pain changed during a session—although minimally—but not over the course of the programs. The pain findings are consistent with previous work (Walter et al., 2019, 2021) and suggest that pain may not globally improve for participants in these interventions or that a single item-indicator may be an insufficient measure of pain. The current study also did not specify where pain was perceived, which is an important specificity in established literature for pain relief (e.g., low back pain vs. fibromyalgia; Naugle et al., 2012), especially considering the complex and reciprocal relationship between depression and pain (Cheng et al., 2022).

Although within-session changes in psychological outcomes have been demonstrated in Surf and Hike Therapy research (Walter et al., 2019, 2023), prior research has not shown enhanced effects of one intervention compared with the other. Specifically, Surf Therapy produced immediate effects on positive affect and depression/anxiety symptoms among active duty service members (Walter et al., 2019), but the immediate effects of Surf Therapy did not differ from Hike Therapy (Walter et al., 2023) or across various recreational sports when compared (Walter et al., 2021). However, results from this study demonstrated that participants in Surf Therapy reported greater changes in pain and increases in positive affect than those in Hike Therapy from pre- to postsession—even when depression severity was taken into account. These between-group differences in within-session changes are novel findings that also corroborate theory. The “blue space” framework posits that water-based activities provide sensory benefits that enhance psychological and functional outcomes (Haeffner et al., 2017; Britton et al., 2020). This suggests that the sights, smells, and sounds inherent to surfing may enrich positive affect through neurological or cognitive changes. Another possibility is that surfing requires a higher degree of skill, and that building proficiency results in greater improvements in positive affect immediately following a session. Surfing also may produce a rush of adrenaline or norepinephrine that temporarily relieves pain (or, here, does not worsen it; Fleischmann et al., 2011), and it requires intense focus that may serve as a reprieve from mental health symptoms in military samples (Caddick and Smith, 2017; Marshall et al., 2020). Although such interpretations may help to explain the observed between-group differences, further research is needed to confirm these mechanisms.

Limitations of this work should be noted. Study participants were U.S. active duty service members with MDD, so results may not generalize to other populations. Although clinician-administered assessments were used to evaluate MDD diagnosis for inclusion and PTSD for descriptive purposes, the specific secondary outcomes for this study were derived from self-report measures and are subject to biases in interpretation and reporting. Preprogram physical activity was measured using the IPAQ-SF, which does not differentiate physical activity done for work or during leisure time—these may have differing effects on psychological health (Harvey et al., 2010). The Surf and Hike Therapy programs evaluated in this randomized controlled trial followed standard operating procedures and program policies. This improved external validity but precluded the ability to empirically control certain components (e.g., optional yoga through only the Surf Therapy Program; discharge policy), although these were statistically controlled for when possible. Similarly, we were unable to establish which program components (e.g., physical activity, natural environment, and social connection) produced the most benefit or whether these produced differential effects by program.

This study also has several strengths that can enhance knowledge about outdoor exercise interventions, including those specific to surfing and hiking. This study was conducted as a randomized controlled trial and evaluated a variety of psychological and functional outcomes that supported prior primary outcome depression results (Walter et al., 2023). Depression outcomes have been more extensively studied in the areas of exercise-based research, so the current study adds to this literature by providing data on other important outcomes, including those that are often comorbid with depression. Furthermore, data were collected at immediate (pre- and postsession), short-term (pre- to postprogram), and longer-term (postprogram to 3-month follow-up) time points, allowing for a more detailed examination of the duration of intervention effects. Data on concurrent treatment were collected and included as variables in statistical models. In sum, this study demonstrated that outdoor exercise interventions can improve an array of psychological and functional outcomes for service members with MDD, and it further supports these interventions as effective adjunctive approaches.

Surf and hike therapies are novel interventions that may be particularly beneficial for military populations, given the emphasis on physical activity and may confer less stigma than traditional treatments (Ben-Zeev et al., 2012; Skopp et al., 2012). These interventions may offer particular benefit to service members with MDD as they offer prescribed opportunities to engage in outdoor activities and social interaction, which may be limited due to the disorder. Both therapies show promise in alleviating symptoms of MDD (Walter et al., 2023), and results from this study extend prior findings by demonstrating that the intervention effects extend to related psychological and functional outcomes, including general anxiety symptoms, positive and negative affect, and social functioning. Further research is needed to explore the mechanisms through which these changes occurred (e.g., exercise, exposure to nature, and social interaction) and to determine the generalizability of results to other populations, which could aid the development and implementation of outdoor exercise programs for military service members and civilians alike.

## Author’s note

KW, KK, and BM-K are employees of the U.S. Government. This work was prepared as part of our official duties. Title 17, U.S.C. §105 provides that copyright protection under this title is not available for any work of the U.S. Government. Title 17, U.S.C. §101 defines a U.S. Government work as work prepared by a military service member or employee of the U.S. Government as part of that person’s official duties.

## Data availability statement

The datasets presented in this article are not readily available because of security protocols and privacy regulations, but they may be made available on reasonable request by the Naval Medical Center San Diego or Naval Health Research Center IRBs (contact phone +1.619.553.8400) or by contacting the corresponding author to facilitate the request. Requests to access the datasets should be directed to KW, kristen.h.walter.civ@health.mil.

## Ethics statement

The studies involving human participants were reviewed and approved by Naval Medical Center San Diego IRB, protocol number NMCSD.2017.0007. The patients/participants provided their written informed consent to participate in this study.

## Author contributions

KW: conceptualization, methodology, investigation, resources, writing—original draft, writing—review and editing, supervision, project administration, and funding acquisition. NO: conceptualization, methodology, formal analysis, investigation, resources, data curation, writing—original draft, writing—review and editing, and project administration. EM: investigation, writing—original draft, and writing—review and editing. TR: writing—review and editing. LG: investigation, resources, data curation, writing—review and editing, and supervision. JB: investigation and writing—review and editing. KK and BM-K: investigation, resources, and supervision. All authors contributed to the article and approved the submitted version.

## Funding

This work was supported by the U.S. Navy Bureau of Medicine and Surgery (work unit no. N1600). The funders had no role in the design, conduct, analysis, or reporting of this trial.

## Conflict of interest

The spouse of KW is an employee of Google LLC and has stock options as part of compensation package. Google LLC owns Fitbit products, which were used in the study for secondary data collection. Fitbit data are not reported in the current manuscript. When published, results are not expected to affect the value of the company’s stock. NO, EM, TR, and LG are employed and JB was employed by Leidos.

The remaining authors declare that the research was conducted in the absence of any commercial or financial relationships that could be construed as a potential conflict of interest.

## Publisher’s note

All claims expressed in this article are solely those of the authors and do not necessarily represent those of their affiliated organizations, or those of the publisher, the editors and the reviewers. Any product that may be evaluated in this article, or claim that may be made by its manufacturer, is not guaranteed or endorsed by the publisher.

## Author disclaimer

The views expressed in this article are those of the authors and do not necessarily reflect the official policy or position of the Department of the Navy, Department of Defense, nor the U.S. Government.
